# Cultivation Method-Driven Aroma Diversification in *Antrodia cinnamomea*: GC-IMS and Bioelectronic Sensors Reveal Distinctive Volatile Fingerprints

**DOI:** 10.3390/foods14162790

**Published:** 2025-08-11

**Authors:** Xiaofeng Ma, Na Zhang, Shiyuan Yu, Tianyu Shi, Shude Yang, Xianhao Cheng, Yongfei Ming, Rui Zhang

**Affiliations:** 1 Yantai Edible and Medicinal Mushroom Technology Innovation Center, Key Laboratory of Molecular Module-Based Breeding of High Yield and Abiotic Resistant Plants in Universities of Shandong, College of Horticulture, Ludong University, Yantai 264025, China; 15684137756@163.com (X.M.); 15610309592@163.com (N.Z.); ysy092266@163.com (S.Y.); sdyang68@126.com (S.Y.); chengxianhao@sohu.com (X.C.); 2 School of Life Science, Ludong University, Yantai 264025, China; 17686023757@163.com

**Keywords:** *Antrodia cinnamomea*, E-nose, E-tongue, gas chromatography–ion mobility spectrometry (GC-IMS), fragrance-emitting volatiles, principal component analysis (PCA)

## Abstract

*Antrodia cinnamomea*, a renowned rare medicinal fungus in China, is rich in active components, exhibiting pharmacological effects, such as liver protection, hypoglycemic activity, and anti-tumor properties. Aiming to address the lack of horizontal comparative studies on volatile components of *A. cinnamomea* under different culture methods and the limitations of traditional detection methods, this study investigated the mycelia of *A. cinnamomea* cultured by solid-state culture (SAC), liquid culture (LAC), and dish culture (DAC). The flavor profiles were comprehensively evaluated using a combination of electronic tongue (E-tongue), electronic nose (E-nose), gas chromatography–ion mobility spectrometry (GC-IMS), and multivariate statistical methods. Results from E-tongue and E-nose showed distinct flavor profiles among the three culture methods. A total of 75 volatile compounds were detected by GC-IMS, among which esters, alcohols, and ketones were the main components, accounting for 62.7%. Partial least squares discriminant analysis (PLS-DA) identified 41 characteristic volatile compounds, and cluster heatmaps and orthogonal partial least squares discriminant analysis (OPLS-DA) further validated the metabolic preferences among culture methods. These findings provide a scientific basis for improving *A. cinnamomea* product quality through targeted flavor enhancement, support the development of standardized functional foods, and establish a flavor fingerprint for authenticity assessment, advancing the high-value utilization of this medicinal fungus.

## 1. Introduction

*Antrodia cinnamomea* (T.T. Chang & W.N. Chou 1995), commonly known as “ox camphor mushroom”, belongs to *Antrodia* P. Karst. 1879, Fomitopsidaceae, Polyporales, Agaricomycetes, Basidiomycota. As a rare medicinal fungus endemic to Taiwan, China, it is renowned as the “forest ruby” [1]. Historically used in traditional folk medicine, *A. cinnamomea* was first recognized by Indigenous Taiwanese communities for its efficacy in alleviating hangovers, liver disorders, and physical exhaustion. These ethnomedical applications have since sparked scientific interest, leading to investigations that unraveled their broader medicinal potential [2].

Wild *A. cinnamomea* grows exclusively in the decayed hollows of *Cinnamomum kanehirae Hay* trunks or on fallen camphor wood [3,4]. Due to scarce hosts and stringent growth requirements, wild populations are critically endangered. Current cultivation methods include log culture, solid-state culture, liquid culture, and dish culture. Log culture involves inoculating *A. cinnamomea* onto camphor or apple wood, but camphor logs are increasingly scarce, and non-camphor substrates require lengthy cultivation (often over two years), failing to meet market demand [5]. Solid-state culture uses oat-based media to mimic natural conditions, offering simplicity but longer cycles [6]. Liquid culture in PDA medium enables rapid mycelial growth via controlled temperature, pH, and aeration, suitable for industrial scale-up [7]. Dish culture employs agar-PDA medium, combining convenience and moderate cycles for visual growth monitoring and morphological studies [8].

Unlike many medicinal fungi, *A. cinnamomea* emits a pronounced and complex fragrance during mycelial growth, characterized by sweet, woody, and fruity notes. This distinctive aroma profile distinguishes it from other fungal species in cultivation. Traditional analytical methods like gas chromatography–mass spectrometry (GC-MS) and gas chromatography–olfactometry (GC-O) require complex pretreatment, struggle to distinguish isomers, and cannot resolve the contribution of mixed volatiles to overall aroma [9]. In contrast, E-sense technology (E-nose and E-tongue) simulates human olfaction and gustation for rapid flavor assessment [10]. Gas chromatography–ion mobility spectrometry (GC-IMS) technology is a powerful technique for separating and sensitively detecting volatile organic compounds [11]. It is characterized by stronger separation ability, higher sensitivity, and easy operation. GC-IMS technology can effectively separate volatile compounds in complex mixtures, operate under atmospheric pressure, and samples require almost no preparation steps [12]. Meanwhile, it can directly identify volatile substances in liquid or solid samples and generate fingerprint patterns without complex post-processing [13]. Moreover, this technology exhibits remarkably high-throughput analysis characteristics, enabling rapid analysis of a large number of samples in a short time and effectively identifying volatile compounds at microgram-level concentrations [14]. These technologies are widely used in environmental and food industry research [15,16,17].

Against this backdrop, this study explicitly puts forward the core research questions: Do the three cultivation methods—solid-state culture (SAC), liquid culture (LAC), and dish culture (DAC)—lead to significant differences in the types, contents, and flavor characteristics of volatile components in *A. cinnamomea*? What are the core features of these differences? Can distinctive volatile compounds be used to establish identification markers for different cultivation methods? Meanwhile, based on the environmental differences among the three cultivation methods, the research hypotheses are proposed: Solid-state culture, due to the complexity of the oat-based medium, may promote the accumulation of characteristic aroma substances such as esters and alcohols; liquid culture, due to the uniform and easily absorbable nutrients, may accelerate the specific synthesis of pyrazines and nitrogen-containing heterocyclic compounds; dish culture, due to the constraints of the agar medium, may result in intermediate levels of ketones and some esters. Additionally, the differences in volatile components among the three cultivation methods can be effectively distinguished, and the characteristic compounds can be screened out by GC-IMS combined with electronic nose/tongue technology.

Existing studies have mainly focused on the analysis of volatile components of *A. cinnamomea* under a single cultivation method. Although they have respectively revealed some characteristics of solid-state, liquid, and dish cultivation, there is a lack of a horizontal comparative framework. For example, Xia et al. only reported the dynamic change process of volatile substances during the solid-state fermentation of *A. cinnamomea*, without involving differences from other cultivation methods [18]; Liu et al. identified 42 volatile compounds in the liquid fermentation of *A. cinnamomea* using GC-MS and GC-O techniques, but did not establish a connection with the solid-state cultivation system [19]. He et al. studied the volatile compounds in *A. cinnamomea* mycelia obtained from PDA solid medium and liquid medium composed of glucose, peptone, and magnesium sulfate, demonstrating that the overall aroma of fermentation products was dominated by mushroom, fruit, and floral scents [20].

This study establishes, for the first time, a comparative framework integrating GC-IMS with electronic nose and tongue technologies to analyze volatile compounds in *A. cinnamomea* under three cultivation systems (SAC, LAC, and DAC), offering a foundational dataset for cultivation-dependent flavor optimization. By identifying key aroma components, this work may help guide cultivation methods to improve the sensory quality of *A. cinnamomea* products. Additionally, correlating flavor profiles with bioactive compounds could assist in developing more consistent functional ingredients. The proposed flavor fingerprint approach also offers a potential tool for basic quality evaluation of different *A. cinnamomea* samples. These findings contribute to ongoing efforts to better characterize and utilize this valuable fungal resource.

## 2. Materials and Methods

### 2.1. Experimental Materials

The *A. cinnamomea* strain was purchased from the Guangdong Provincial Microbial Culture Collection Center (GIM No.5.530) and preserved on PDA slant medium (composition: 200 g potato(Zhenhua Liangfan Supermarket Co., Ltd., Yantai, Shandong, China), 20 g glucose, 1 g potassium dihydrogen phosphate, 0.5 g magnesium sulfate, 3 g yeast extract powder, and 20 g agar dissolved in 1 L distilled water), All drugs except potatoes were purchased from Macklin Biochemical Technology Co., Ltd., Shanghai, China. Molecular identification and taxonomic analysis of the target strain were performed using PCR amplification of the internal transcribed spacer (ITS) region of ribosomal DNA, combined with sequencing analysis and phylogenetic methods.

The samples in this study were *A. cinnamomea* mycelia obtained through three culture methods, with the culture media and conditions shown in Table 1. The obtained mycelia were dried in an oven at 40 °C (DHT-450A, Daohan Industrial Co., Ltd., Shanghai, China), ground, sieved (pore size 0.425 mm), and stored at 4 °C for subsequent use.

### 2.2. E-Tongue Analysis

Taste analysis was performed using a 10.10.5.41 electronic tongue (Shanghai Baosheng Industrial Development Co., Ltd., Shanghai, China). 2.0 g of *A. cinnamomea* samples from each of the three culture methods were separately weighed and added to 20 mL of distilled water. The mixtures were equilibrated in a water bath at 50 °C for 60 min, then cooled and filtered through a 0.22 μm membrane to obtain clear filtrates. The filtrates were transferred to specialized electronic tongue cups for detection, with a measurement time of 140 s and a magnification factor of 100. Distilled water was used as a blank control to correct background signals and eliminate environmental interference. Each sample was analyzed in 5 technical replicates.

### 2.3. E-Nose Analysis

Odor profile analysis was performed using a 10.10.5.42 electronic nose (Shanghai Baosheng Industrial Development Co., Ltd., Shanghai, China). 1 g of *A. cinnamomea* samples from each culture method were placed into 40 mL headspace vials, sealed with PTFE-sealed caps, and equilibrated in a 50 °C water bath for 60 min. The headspace gases were aspirated for detection by the electronic nose system, which was set with a detection time of 60 s, a cleaning time of 120 s, and a gas flow rate of 1 L/min. An empty sample bottle was used as a blank control to correct the background signal and eliminate environmental interference. Each sample was analyzed in 5 technical replicates. The response types of the sensor probes to different compounds are shown in Table 2.

### 2.4. GC-IMS Analysis

Volatile compound analysis was performed using GC - IMS (Haineng Future Technology Group Co., Ltd., Jinan, China). Headspace Sampling Conditions: Accurately weigh 2.0 g of dried mycelium sample that has been ground and sieved (0.425 mm), and place it in a 20 mL headspace sampling vial. After sealing the sampling vial, incubate it in a constant—temperature water bath at 60 °C for 15 min with a rotation speed of 500 r/min to ensure the sufficient release of volatile components. An 800 μL headspace injection is adopted (in splitless mode), and the temperature of the injection needle is set to 80 °C.

GC-IMS Conditions: An MXT-WAX capillary column (15 m × 0.53 mm × 1 μm) was used. The column temperature was set at 60 °C, with an analysis time of 30 min. The migration tube temperature was 45 °C. Nitrogen with a purity of 99.99% served as the carrier gas, with an initial flow rate of 2 mL/min. The linear pressure program was as follows: 2 mL/min for 2 min, increased to 10 mL/min for 8 min, then to 100 mL/min for 10 min, and finally to 150 mL/min for 10 min. Nitrogen was used as the drift gas at a flow rate of 150 mL/min. Each spectrum was averaged over 12 scans, and all samples were analyzed in three technical replicates. An empty sample bottle was used as a blank control to correct the background signal and eliminate environmental interference.

### 2.5. Data Collection and Statistical Analysis

GC-IMS data were collected and analyzed using the instrument’s built-in Vocal0.4.03 software, with qualitative analysis performed against the software’s internal database. The Reporter plugin in Vocal0.4.03 was utilized to generate differential maps, while the Gallery Plot plugin was employed to create volatile compound fingerprint profiles. Radar charts were processed using SIMCA14.1 software, and principal component analysis (PCA) along with partial least squares discriminant analysis (PLS-DA) were conducted via the bioinformatics online platform (https://www.bioinformatics.com.cn/, accessed on 14 April 2025), which was also used to generate differential compound heatmaps.

## 3. Results

### 3.1. E-Tongue Analysis

**Figure 1:**
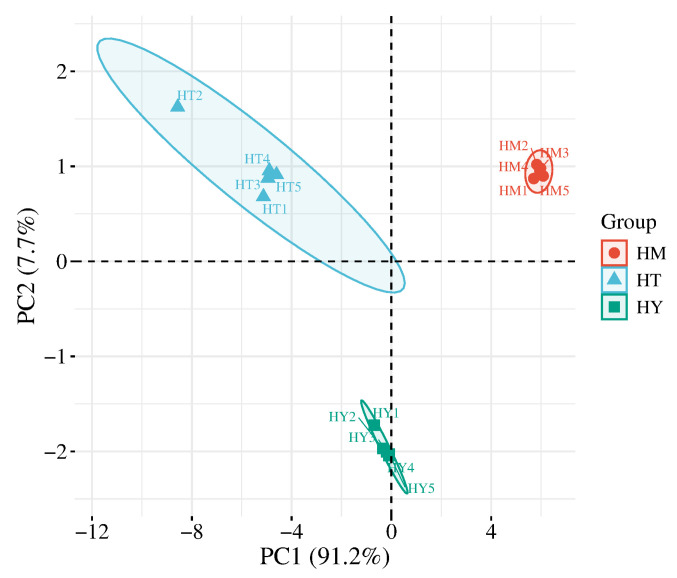
PCA score plot of E-tongue analysis. HY—Solid-state cultured mycelia (SAC); HT—Liquid cultured mycelia (LAC); HM—Plate cultured mycelia (DAC) (the same below).

Principal component analysis (PCA) is a statistical method that transforms multiple variables into a few uncorrelated principal components through linear transformation, thereby highlighting differences between samples [21]. As shown in Figure 1, the variance contribution rates of PC1 and PC2 were 91.2% and 7.7%, respectively, with a cumulative variance contribution rate of 98.9%. This indicates that these two principal components can represent most of the information in the samples, with PC1 playing a dominant role in distinguishing sample differences. The three sample groups were clearly separated in the coordinate space, demonstrating significant differences among *A. cinnamomea* cultivated by different methods and confirming the effectiveness of E-tongue technology in distinguishing them.

### 3.2. E-Nose Analysis

**Figure 2:**
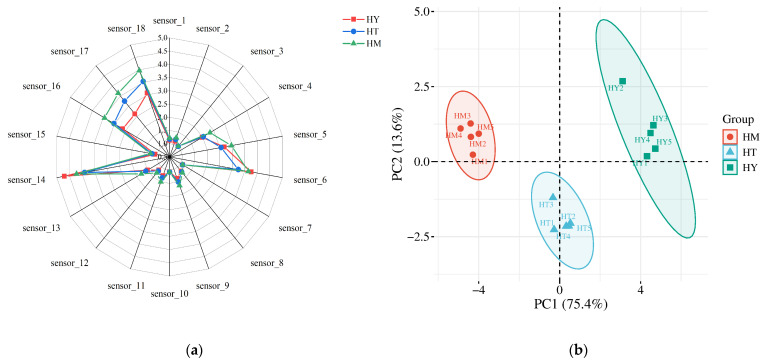
E-nose analysis of *A. cinnamomea* samples: (**a**) Radar chart showing sensor response patterns; (**b**) PCA score plot.

The volatile components were analyzed using an electronic nose, with the corresponding radar chart and PCA results presented in Figure 2a and Figure 2b, respectively. Figure 2a displays the response values of different e-nose sensors to specific volatile compounds, where each sensor demonstrates cross-sensitivity to particular chemicals [22]. The shape and area of the radar chart reflect distinct profiles among samples. Results revealed significantly higher response intensities to sensors S6, S14, S16, S17, and S18 for *A. cinnamomea* from different cultivation methods, with observable variations among groups. This indicates these five sensors effectively discriminated flavor profiles across cultivation methods. In samples of solid-state cultivation (SAC), the signals of sensor S6 (responsive to toluene, acetone, etc.) and sensor S18 (responsive to acetone and ethanol) were significantly enhanced. This is consistent with the increased contents of esters (such as Linalyl butanoate) and alcohols (such as 3-Methyl-3-buten-1-ol) detected by GC-IMS, indicating that these compounds are the main contributors to the “fruity-floral” characteristics of SAC samples. In contrast, other sensors showed minimal response differences with nearly overlapping signals, suggesting minor compositional variations in detected components. Further PCA of odor profiles (Figure 2b) demonstrated variance contribution rates of 75.4% (PC1) and 13.6% (PC2), yielding a cumulative 89% variance explained. The clear spatial separation without overlap among samples from different cultivation methods confirms statistically significant differences in their odor characteristics.

### 3.3. GC-IMS Analysis

#### 3.3.1. Fingerprint Analysis of Volatile Compounds in *A. cinnamomea* with Different Culture Methods

**Figure 3:**
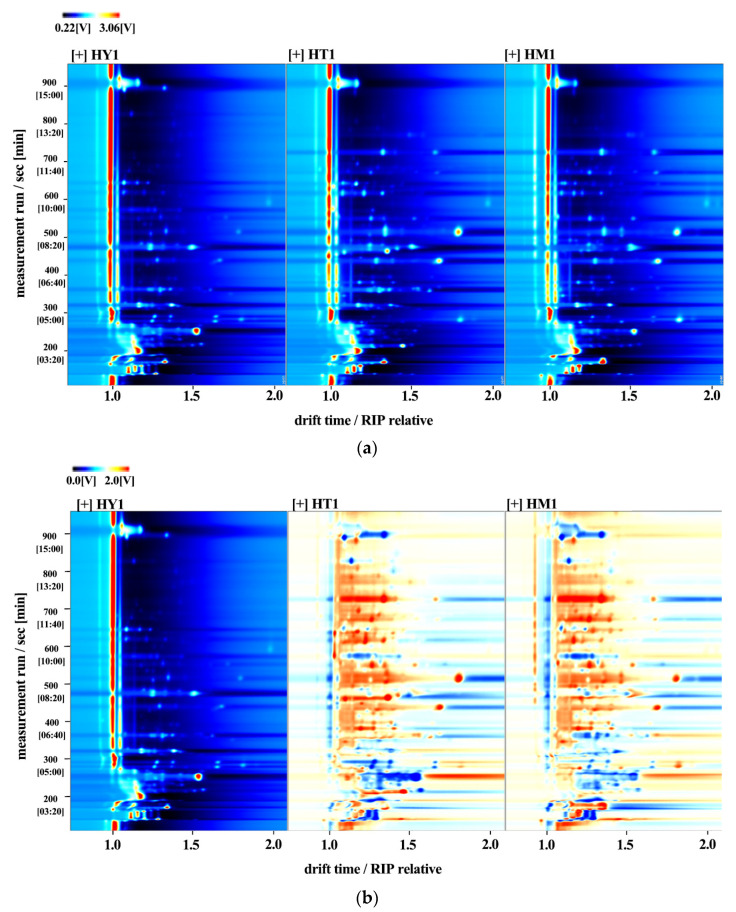
GC-IMS analysis of *A. cinnamomea* mycelia under different culture methods: (**a**) Two-dimensional spectrum (color gradient represents signal peak intensity of compounds: red indicates high intensity, white indicates low intensity, with darker colors corresponding to greater intensity); (**b**) Differential comparison model between culture methods.

Gas chromatography–ion mobility spectrometry (GC-IMS) was further used to qualitatively analyze and compare volatile compounds in *A. cinnamomea* under different culture methods. By normalizing the ion migration time and the position of the reaction ion peak (RIP), the results were visualized as a two-dimensional map in Figure 3a, which covered all compounds in the samples. The volatile compound compositions of *A. cinnamomea* under different culture methods showed similarities. Single points on the right side of the reaction ion peak represented volatile compounds extracted from the samples [23]. Most signals appeared within the range of retention time (100–800 s) and drift time (1–2 s). In the spectrum, the red signals (high intensity) are mainly concentrated in the region with a retention time of 100–300 s and a drift time of 1.2–1.5 s, corresponding to ester and alcohol compounds (such as Linalyl butanoate and 3-Methyl-3-buten-1-ol). Among them, the signal intensity of SAC in this region is significantly higher than that of LAC and DAC, which is consistent with the result that SAC has the highest ester content in the subsequent fingerprint. Multiple peaks (such as the monomer “M” and dimer “D” of Butyl butyrate) appearing at the same retention time (e.g., 300–400 s) indicate that some compounds exist in polymerized forms, which is consistent with the separation characteristics of GC-IMS for small-molecule volatile substances.

A difference comparison model was employed to assess sample discrepancies in Figure 3b: using the topographic map of HY1 as a control, topographic maps of other samples were subtracted from the control to identify differences among the three sample groups. A white background indicated consistent volatile compounds, while red and blue colors represented concentrations higher or lower than the control, respectively.

**Figure 4:**
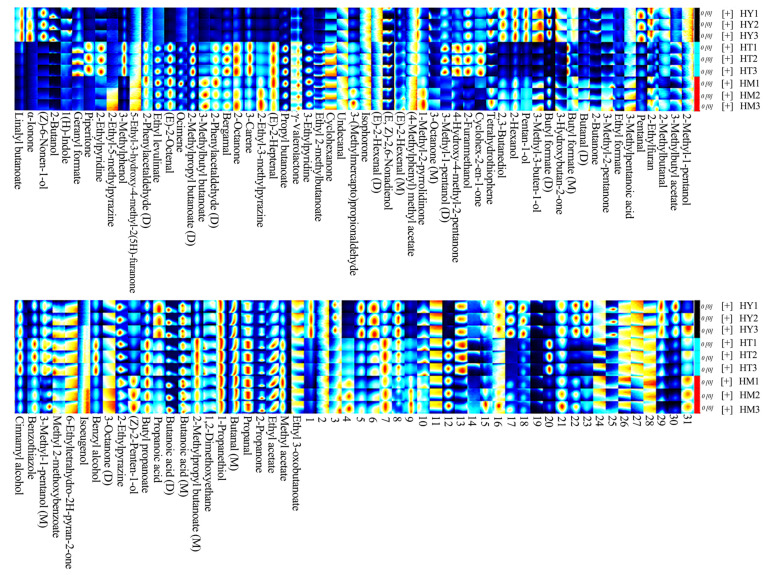
GC-IMS volatile compound fingerprint spectra of *A. cinnamomea* mycelia under different culture methods.

Although topographic maps provided trends and information about detected substances, accurately identifying volatile compounds remained challenging. To deeply analyze the differential components of volatile compounds in *A. cinnamomea* under different culture methods, corresponding fingerprint maps were generated using built-in software plugins (Figure 4). In the fingerprint map, each row represented a sample, and each column represented a volatile substance. Each point in the map represented a specific substance, where the color area and brightness of volatile compounds indicated their concentrations-the brighter the color, the higher the content [24]. Monomers were labeled “M” and dimers “D” behind some substance names, while unidentified peaks were numbered.

As shown in Figure 4, volatile compounds such as esters, alcohols, and ketones were detected in *A. cinnamomea* across all three culture methods, with esters, alcohols, and ketones being the main components. However, their compositions and contents varied significantly among culture methods. In SAC, compounds such as Linalyl butanoate, α-Ionone, (Z)-6-Nonen-1-ol, and 2-Butanone were detected, and the contents of compounds like 3-Methyl-3-buten-1-ol and Propanoic acid were significantly higher than those in the other two culture methods. This may be related to the complex matrix of the oat-based medium in SAC. The results of this study are consistent with those of Xia’s research, further indicating that the complex components of the oat matrix may activate related metabolic pathways, leading to increased contents of these substances [18]. Piperitone and 2-Ethylpyridine were only detected in LAC, presumably because the uniform nutrition and rapid growth characteristics of liquid culture promoted the synthesis of specific heterocyclic and nitrogen-containing compounds. Liu et al. mentioned in their study on the aroma components of *A. cinnamomea* in liquid culture that the rapid transfer and uniform distribution of nutrients in the liquid environment can promote the synthesis of specific terpenoids and nitrogen-containing compounds [19]. The results of LAC in this study are consistent with theirs, confirming the promoting effect of liquid culture conditions on the synthesis of such compounds. In DAC, the signal intensities of certain esters (e.g., Butyl formate) and aldehydes (e.g., Pentanal) were at an intermediate level. This is related to the physical properties of the agar medium that affect metabolite accumulation, which is consistent with the research conclusions of He et al [20]. These differences indicated that different culture methods led to the diversity in the composition and content of volatile compounds by influencing microbial metabolic pathways, with SAC demonstrating better performance in the richness and intensity of characteristic aroma substances. It is worth noting that in SAC, α-Ionone, a key aroma compound with hepatoprotective activity, and triterpenes share a common isopentenyl pyrophosphate precursor [25]. In addition, Methyl cinnamate, a type of ester, not only has a unique fruity and floral aroma but also exhibits certain anti-inflammatory activity, which can inhibit the release of inflammatory factors [26]. These findings provide preliminary clues for the association between aroma and medicinal efficacy.

#### 3.3.2. Qualitative Results of Volatile Compounds in *A. cinnamomea* with Different Culture Methods

Six standard n-ketones (2-butanone, 2-pentanone, 2-hexanone, 2-heptanone, 2-octanone, 2-nonanone) were used as external references to obtain retention indices of volatile compounds by comparing their ion migration times and retention times [27]. Qualitative analysis of volatile components in samples was achieved by matching data with the IMS database. A total of 75 volatile compounds (monomers and dimers counted once) were identified from the samples, as shown in Table 3, including 19 esters, 15 alcohols, 13 ketones, 11 aldehydes, nine heterocyclic compounds, three acids, two phenols, two alkenes, one ether, with 31 compounds remaining unidentified. The molecular weights of the compounds ranged from 58.1 (e.g., 2-Propanone) to 224.3 (e.g., Linalyl butanoate), reflecting the structural complexity and diversity of the compounds. Results showed that volatile compounds in *A. cinnamomea* were mainly composed of esters, alcohols, and ketones.

Most of the identified 75 volatile compounds exhibited characteristic aromas. C8 aliphatic compounds are major contributors to mushroom flavor [28]. Esters (e.g., Methyl 2-methoxybenzoate), formed by the reaction of acids and alcohols, possess unique fruity and floral odors due to their ester groups and methoxy structures, which influence both odor and chemical stability [29]. Alcohols such as 3-Methyl-1-pentanol impart mellow aromas to *A. cinnamomea*, with their hydroxyl group positions and carbon chain structures affecting volatility and odor [30]. Pyrazines, including 2-Ethyl-3-methylpyrazine, are heterocyclic compounds containing two nitrogen atoms, contributing roasted and nutty notes. These compounds act synergistically to form the complex and diverse flavor profile of *A. cinnamomea*.

#### 3.3.3. Partial Least Squares Discriminant Analysis (PLS-DA) of Volatile Compounds in *A. cinnamomea* with Different Culture Methods

**Figure 5:**
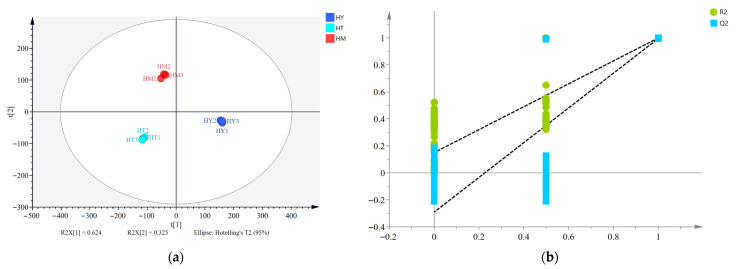
(**a**) Partial least squares discriminant analysis (PLS-DA); (**b**) permutation test.

PLS-DA is recognized as a highly effective method for sample classification and discriminant model construction [31]. A PLS-DA model was established to identify differences in volatile compounds among *A. cinnamomea* samples, as shown in Figure 4. The experimental results showed no overlap between samples, indicating that the PLS-DA model could effectively distinguish the data. Additionally, the model’s principal component regression coefficients were R^2^X = 0.949, R^2^Y = 0.998, and Q^2^ = 0.993, all of which were greater than 0.5, confirming that the PLS-DA model was suitable for prediction. To prevent overfitting of the PLS-DA model and ensure its predictive accuracy for unknown data samples, a 200-time permutation test was used to evaluate the model’s stability and predictive ability (Figure 5). The R^2^ and Q^2^ values on the far right were both higher than 0.85, significantly higher than those on the left, and the Q^2^ regression line had a negative intercept, indicating that the PLS-DA model was stable and free from overfitting.

**Figure 6:**
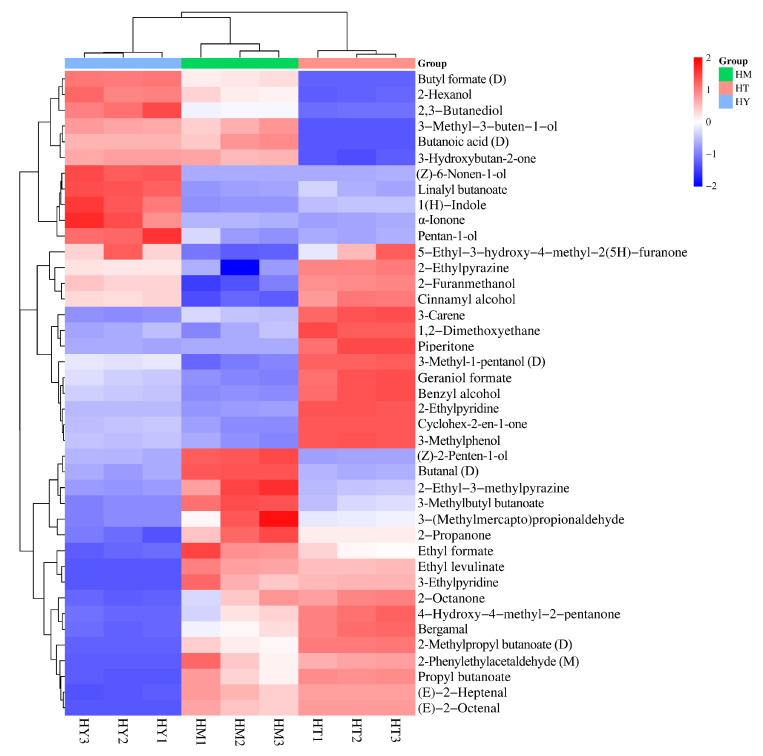
Cluster heatmap of differential volatile compounds in *A. cinnamomea* under different culture methods.

The importance of variables in the PLS-DA model is typically determined by VIP values. It has been reported that variables with VIP values > 1 and *p* < 0.05 have the strongest influence on the model [32,33]. Volatile compounds in different samples were screened using VIP values, and a total of 41 differential volatile compounds were identified, including 11 alcohols, nine esters, seven heterocyclic compounds, six ketones, three aldehydes, three alkenes, and two terpenes. The top five volatile compounds in terms of VIP value are 2-Furanmethanol, (Z)-2-Penten-1-ol, 3-Methylphenol, Ethyl levulinate, and (Z)-6-Nonen-1-ol, respectively. Their VIP values are all greater than 1.5, and they are the core markers for distinguishing the volatile components of *A. cinnamomea* under different cultivation methods. These compounds, due to different cultivation methods (SAC, LAC, and DAC) and through metabolic conversions (such as the metabolism of furans in oat medium, rapid carbon metabolism in a liquid environment, etc.), exhibit sensory differences like bread-coffee aroma and floral notes. Their metabolic pathways are related to the secondary metabolism of *A. cinnamomea*, providing clues for exploring the association between flavor and efficacy. The differences were clearly visualized through a cluster heatmap (Figure 6). The distribution of different colors in the map intuitively reflects the content differences of volatile compounds in each sample, where red represents higher content and blue represents lower content. Meanwhile, samples from different culture methods showed distinct clustering in the heatmap, indicating differences in the composition of volatile compounds in *A. cinnamomea* under different culture methods.

#### 3.3.4. Analysis of Significantly Different Volatile Compounds in Pairwise Comparisons

**Figure 7:**
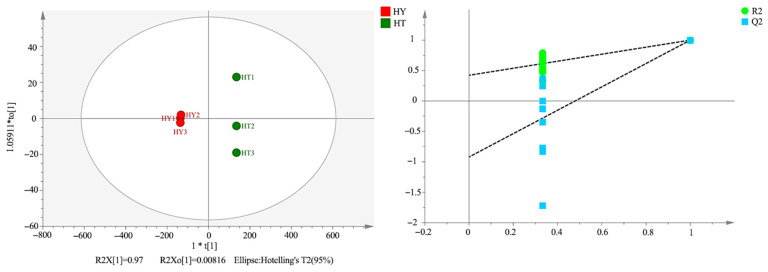
Orthogonal Partial Least Squares Discriminant Analysis (OPLS-DA) and Permutation Test Plot of SAC and LAC.

To further verify the differences in volatile compounds among the three cultivation methods, this study employed the OPLS-DA model to conduct pairwise comparisons between the groups of SAC vs. LAC, LAC vs. DAC, and SAC vs. DAC. Given that clear separation patterns were observed among all groups, the results of the core comparison group (SAC vs. LAC) are presented in Figure 7. This group comparison highlights the most prominent metabolic differentiation between the solid-state cultivation and liquid-state cultivation methods, which have significant differences in medium properties and growth kinetics. The model exhibits strong discriminative ability, and there is no overlap of samples in the score plot, confirming the existence of significant differences in their volatile component profiles. Meanwhile, the reliability of the established model was verified through 200 permutation tests. The results obtained from LAC vs. DAC and SAC vs. DAC are consistent with the overall trend observed in the core comparison group.

**Figure 8:**
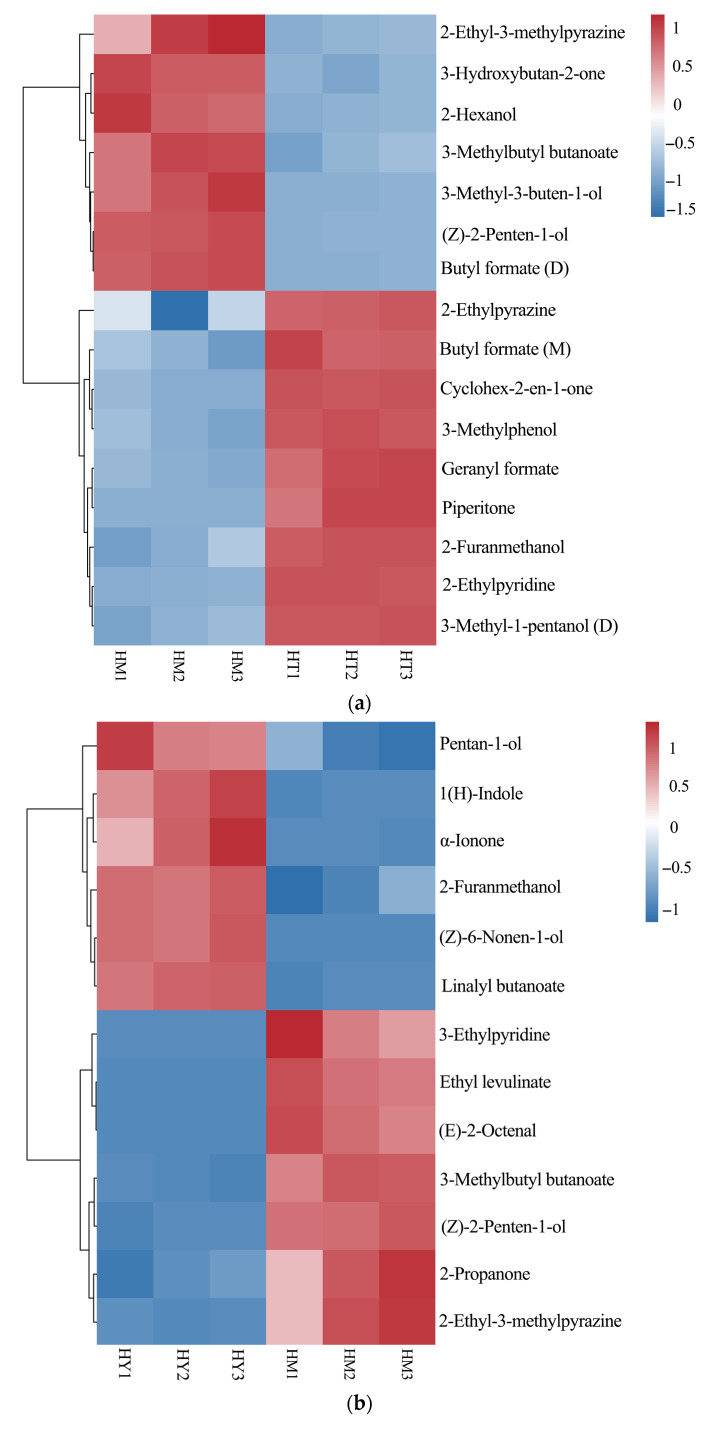

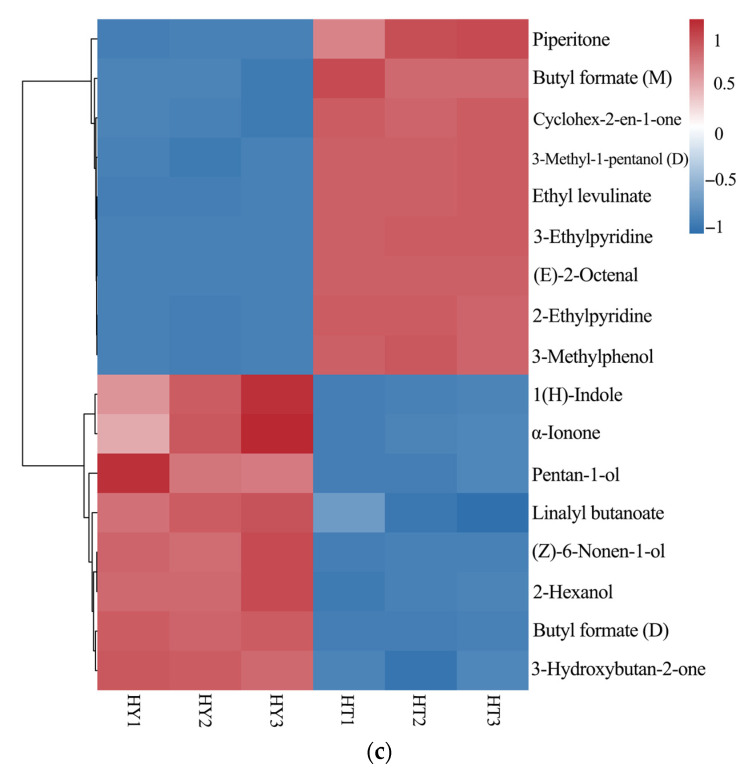
Heatmap of significantly different compounds from pairwise comparisons of three sample groups: (**a**) LAC vs. DAC; (**b**) SAC vs. DAC; (**c**) SAC vs. LAC.

Based on the pairwise OPLS-DA model, metabolites with significant differences were screened under the criteria of *p* < 0.05 and VIP > 1, and a heatmap was drawn as shown in Figure 8. The analysis revealed 16 differential compounds between DAC and LAC, 13 between SAC and DAC, and 17 between SAC and LAC. In the LAC culture method, pyrazines (e.g., 2-Ethyl-3-methylpyrazine) and alcohols (e.g., 2-Hexanol) were relatively upregulated, possibly because the culture conditions favored the activity of related synthetic enzymes. Under the DAC culture method, ketones (e.g., Cyclohex-2-en-1-one) and esters (e.g., 3-Methylbutyl butanoate) were significantly upregulated, likely due to the positive effects of environmental factors on the accumulation of synthetic precursors and metabolic regulation.In the SAC culture method, pyridines (e.g., 3-Ethylpyridine) and aldehydes (e.g., (E)-2-Octenal) showed higher contents, which might be attributed to the influence of special substrates or growth regulators on related metabolic pathways. 

The heatmap results demonstrates distinct differences in the composition and content of volatile compounds among sample groups, indicating that different culture methods affect the formation and accumulation of volatile compounds in *A. cinnamomea*. These differences may reflect variations in metabolic pathways or regulatory mechanisms of volatile compounds across sample groups, providing clues for further exploration of the formation mechanism of flavor substances in *A. cinnamomea*.

## 4. Conclusions

This study innovatively integrates multiple technologies including electronic tongue (E-tongue), electronic nose (E-nose), and gas chromatography–ion mobility spectrometry (GC-IMS), combined with multivariate statistics such as principal component analysis (PCA) and partial least squares discriminant analysis (PLS-DA). It conducts, for the first time, a systematic and comprehensive evaluation of the flavor components of *A. cinnamomea* under different cultivation methods, breaking through the research limitations of previous single technologies or a small number of combined methods. Based on electronic tongue and electronic nose technologies, the overall flavor profile differences of *A. cinnamomea* under different cultivation methods were systematically captured and analyzed in an integrated manner. The electronic tongue focuses on differences in the taste dimension, while the electronic nose captures the differentiation of olfactory characteristics. Together, they elucidate that solid-state, liquid, and plate cultivation methods, by regulating the synthesis of taste-active and aroma substances, lead to differences in the sensory characteristics of *A. cinnamomea* at the level of “taste type combination-aroma profile”. A total of 75 volatile compounds were identified by GC-IMS technology, among which esters, alcohols, and ketones were the main components, accounting for 62.7%. Compounds such as Linalyl butanoate (fruity aroma) and α-Ionone (floral aroma) endowed *A. cinnamomea* with complex flavor characteristics.

Meanwhile, 41 characteristic differential compounds were screened out by PLS-DA, and combined with cluster heat map analysis, which provided a powerful support for comparing and distinguishing the differences in volatile compounds of *A. cinnamomea* under different cultivation methods. Among them, SAC showed better performance in the richness and intensity of characteristic aroma substances. Through orthogonal partial least squares discriminant analysis, the differential volatile compounds among various cultivation methods were further analyzed. LAC preferred to accumulate pyrazines and alcohols, DAC tended to accumulate ketones and esters, while SAC accumulated more pyridines, aldehydes, and some alcohols.

Notably, 31 compounds remained unidentified, though their distribution varied among culture methods based on experimental data. Future research could optimize conditions (e.g., improving column separation and mass spectrometry sensitivity/resolution) and integrate high-resolution nuclear magnetic resonance to characterize these unassigned compounds.

The GC-IMS fingerprint established in this study features advantages of simple sample preparation, rapid analysis, and high sensitivity. Combined with the sensory simulation capabilities of the electronic tongue and electronic nose, it can provide a precise tool for flavor quality control of *A. cinnamomea* and its derivative products. In the development of functional foods, based on the clear flavor differences identified in this study, cultivation methods can be directionally regulated to meet product flavor requirements. For instance, *A. cinnamomea* cultivated by SAC, with its high aroma richness, is more suitable for high-end natural spices and characteristic functional foods. In the pharmaceutical field, the differences in volatile substances of *A. cinnamomea* under different culture methods (such as pyrazines in LAC and pyridines in SAC) can provide a basis for the synergistic optimization of the efficacy and flavor of extracts.

This study systematically clarifies the mechanism by which different culture methods affect the volatile components of *A. cinnamomea*, not only laying a solid theoretical foundation for the in-depth development of *A. cinnamomea* in the fields of functional foods, natural spices, and pharmaceuticals, but also its methodological system of “multi-technology integration + multivariate statistical analysis” can be extended to flavor research on other edible and medicinal fungi. This will facilitate the precise development and quality control of natural product resources, promoting the industry to upgrade from experience-oriented to technology-driven precision.

## Figures and Tables

**Table 1:** Culture Medium and Culture Conditions.

Cultivation Method	Culture Medium and Conditions
Solid-state cultivation (SAC)	Oat: water = 1:2, natural pH, cultivated at 26 °C for 60 days
Liquid-state cultivation (LAC)	200 g potato, 20 g glucose, 1 g potassium dihydrogen phosphate, 0.5 g magnesium Sulfate, 3 g yeast extract powder, natural pH, cultivated at 26 °C with a rotation speed of 120 r/min for 14 days
Dish-type cultivation (DAC)	200 g potatoes, 20 g glucose, 20 g agar powder, 1 g potassium dihydrogen phosphate, 0.5 g magnesium sulfate, 3 g yeast extract powder, natural pH, cultivated at 26 °C for 25 days

**Table 2:** Responses of electronic nose sensors to different substances.

Sensor	Responding Substances	Sensor	Responding Substances
S1	Propane, Smoke	S10	Hydrogen
S2	Alcohol, Smoke, Isobutane, Formaldehyde	S11	Liquefied Gas, Alkanes
S3	Ozone	S12	Liquefied Gas, Methane
S4	Hydrogen Sulfide	S13	Methane
S5	Ammonia	S14	Combustible Gas, Smoke
S6	Toluene, Acetone, Ethanol, Hydrogen	S15	Smoke, Isobutane
S7	Methane, Natural Gas, Biogas	S16	Sulfide
S8	Liquefied Gas	S17	Nitride
S9	Toluene, Formaldehyde, Benzene, Alcohol, Acetone	S18	Acetone, Ethanol

**Table 3:** Qualitative results of volatile compounds in *A. cinnamomea* under different culture methods.

Count	Compound	CAS #	Formula	MW	RI	Rt [s]	Dt [a.u.]	Aroma Characteristic
1	Isoeugenol	C97541	C_10_H_12_O_2_	164.2	1454.5	1670.115	1.30445	Sweet and spicy, clove-like
2	Linalyl butanoate	C78364	C_14_H_24_O_2_	224.3	1419.4	1547.341	1.23106	Floral, fruity, sweet
3	α-Ionone	C127413	C_13_H_20_O	192.3	1419.3	1546.983	1.5014	Floral
4	Methyl 2-methoxybenzoate	C606451	C_9_H_10_O_3_	166.2	1305.5	1208.219	1.23242	Fruity and floral
5	Undecanal	C112447	C_11_H_22_O	170.3	1306.1	1209.78	1.60863	Soapy
6	Cinnamyl alcohol	C104541	C_9_H_10_O	134.2	1302	1199.055	1.08834	Cinnamon-like spice, floral
7	1(H)-Indole	C120729	C_8_H_7_N	117.2	1279.6	1142.099	1.15998	Spicy
8	3-Methylphenol	C108394	C_7_H_8_O	108.1	1261.5	1098.096	1.12095	Medicinal, woody, leathery, phenolic aldehyde
9	Geranyl formate	C105862	C_11_H_18_O_2_	182.3	1266.9	1111.099	1.22086	Floral, fruity
10	Piperitone	C89816	C_10_H_16_O	152.2	1259.3	1092.781	1.28315	Herbaceous
11	(4-Methylphenyl) methyl acetate	C2216457	C_10_H_12_O_2_	164.2	1275	1130.658	1.48133	Floral
12	Benzothiazole	C95169	C_7_H_5_NS	135.2	1206.3	973.999	1.15162	Rubber-like, nutty
13	5-Ethyl-3-hydroxy-4-methyl-2(5H)-furanone	C698102	C_7_H_10_O_3_	142.2	1206.6	974.59	1.46926	Sweet, fruity, caramel-like
14	(E, Z)-2,6-Nonadien-1-ol	C28069729	C_9_H_16_O	140.2	1165	890.372	1.15638	Violet leaf
15	(Z)-6-Nonen-1-ol	C35854865	C_9_H_18_O	142.2	1167.8	895.744	1.33275	Muskmelon
16	6-Ethyltetrahydro-2H-pyran-2-one	C3301904	C_7_H_12_O_2_	128.2	1164	888.401	1.59717	Coconut oil, earthy
17	Isophorone	C78591	C_9_H_14_O	138.2	1114.4	797.615	1.77656	Woody, camphoraceous, and musty
18	(E)-2-Octenal	C2548870	C_8_H_14_O	126.2	1071.1	726.127	1.33062	Fresh cucumber, banana
19	Ethyl levulinate	C539888	C_7_H_12_O_3_	144.2	1071.7	726.956	1.6537	-
20	Bergamal	C106729	C_9_H_16_O	140.2	1051.6	695.941	1.17552	Muskmelon with floral notes
21	(E)-Ocimene	C13877913	C_10_H_16_	136.2	1038.3	676.172	1.66822	Floral
22	2-Phenylacetaldehyde (M)	C122781	C_8_H_8_O	120.2	1036	672.754	1.2547	Rose, clover
23	2-Phenylacetaldehyde (D)	C122781	C_8_H_8_O	120.2	1036.3	673.157	1.53661	Rose, clover
24	3-Methylbutyl butanoate	C109193	C_9_H_18_O_2_	158.2	1035.2	671.55	1.38177	Fruity, sweet, pineapple-like
25	Benzyl alcohol	C100516	C_7_H_8_O	108.1	1014.2	641.684	1.16795	Sweet, floral, fruity
26	1-Methyl-2-pyrrolidinone	C872504	C_5_H_9_NO	99.1	1016	644.121	1.10704	-
27	2-Octanone	C111137	C_8_H_16_O	128.2	996.7	617.726	1.32846	Earthy, weedy
28	3-Carene	C13466789	C_10_H_16_	136.2	997.2	618.388	1.2144	Citrus
29	2-Ethyl-3-methylpyrazine	C15707230	C_7_H_10_N_2_	122.2	996.8	617.835	1.16281	Roasted, nutty
30	3-Octanone (M)	C106683	C_8_H_16_O	128.2	978.4	581.395	1.3092	Herbaceous, musty, mushroom-like
31	3-Octanone (D)	C106683	C_8_H_16_O	128.2	978.2	581.082	1.71545	Herbaceous, musty, mushroom-like
32	2-Ethyl-5-methylpyrazine	C13360640	C_7_H_10_N_2_	122.2	984.4	593.014	1.20065	Coffee bean, nutty
33	(E)-2-Heptenal	C18829555	C_7_H_12_O	112.2	961.9	550.356	1.25845	Fruity
34	2-Methylpropyl butanoate (M)	C539902	C_8_H_16_O_2_	144.2	943.3	517.252	1.33593	Fruity
35	2-Methylpropyl butanoate (D)	C539902	C_8_H_16_O_2_	144.2	942	515.106	1.80487	Fruity
36	2-Ethylpyrazine	C13925003	C_6_H_8_N_2_	108.1	917.4	474.695	1.51856	Nutty
37	Cyclohex-2-en-1-one	C930687	C_6_H_8_O	96.1	914.9	470.716	1.41778	Roasted
38	2-Ethylpyridine	C100710	C_7_H_9_N	107.2	909.3	461.994	1.09344	Grass-like
39	Butyl propanoate	C590012	C_7_H_14_O_2_	130.2	893.7	438.659	1.29013	-
40	3-(Methylthio)propionaldehyde	C3268493	C_4_H_8_OS	104.2	893.8	438.789	1.39152	Vegetable
41	Propyl butanoate	C105668	C_7_H_14_O_2_	130.2	893.7	438.605	1.67308	Fruity
42	(E)-2-Hexenal (M)	C6728263	C_6_H_10_O	98.1	874.6	412.448	1.17875	Fruity
43	(E)-2-Hexenal (D)	C6728263	C_6_H_10_O	98.1	874.3	412.021	1.52814	Fruity
44	3-Methylbutyl acetate	C123922	C_7_H_14_O_2_	130.2	874.4	412.193	1.29612	Sweet, banana-like, fruity
45	Ethyl 2-methylbutanoate	C7452791	C_7_H_14_O_2_	130.2	853.7	385.513	1.23938	Fruity
46	3-Methyl-1-pentanol (M)	C589355	C_6_H_14_O	102.2	834	361.798	1.31184	Spicy, wine-like, cocoa-like
47	3-Methyl-1-pentanol (D)	C589355	C_6_H_14_O	102.2	832.2	359.803	1.61025	Dry wine-like, cocoa-like
48	4-Hydroxy-4-methyl-2-pentanone	C123422	C_6_H_12_O_2_	116.2	835.7	363.877	1.54105	-
49	2-Furanmethanol	C98000	C_5_H_6_O_2_	98.1	833.8	361.585	1.36965	Bread and coffee
50	Tetrahydrothiophene	C110010	C_4_H_8_S	88.2	820.8	346.819	1.30684	Chinese cabbage
51	2-Methyl-1-pentanol	C105306	C_6_H_14_O	102.2	822.4	348.601	1.59109	-
52	Butanoic acid (D)	C107926	C_4_H_8_O_2_	88.1	801.4	325.822	1.39448	Dairy-like, buttery, fruity
53	Butanoic acid (M)	C107926	C_4_H_8_O_2_	88.1	799.7	324.037	1.17068	Dairy-like, buttery, fruity
54	2-Hexanol	C626937	C_6_H_14_O	102.2	766.5	291.668	1.27341	Wine-like
55	2,3-Butanediol	C513859	C_4_H_10_O_2_	90.1	766.5	291.695	1.36916	-
56	3-Methyl-2-pentanone	C565617	C_6_H_12_O	100.2	754.3	280.786	1.18103	-
57	Pentan-1-ol	C71410	C_5_H_12_O	88.2	752	278.827	1.2556	Fermentation
58	(Z)-2-Penten-1-ol	C1576950	C_5_H_10_O	86.1	750	277.096	1.45179	Cherry narcissus
59	Butyl formate (M)	C592847	C_5_H_10_O_2_	102.1	717.3	250.238	1.19881	Fruity
60	Butyl formate (D)	C592847	C_5_H_10_O_2_	102.1	708.2	243.275	1.53149	Fruity
61	3-Hydroxybutan-2-one	C513860	C_4_H_8_O_2_	88.1	729.6	259.981	1.33219	-
62	3-Methyl-3-buten-1-ol	C763326	C_5_H_10_O	86.1	727.2	258.1	1.4198	Fruity
63	2-Ethylfuran	C3208160	C_6_H_8_O	96.1	707	242.317	1.30749	Malt, coffee, nuts
64	Propanoic acid	C79094	C_3_H_6_O_2_	74.1	692.1	231.366	1.26102	Pungent acidity
65	Pentanal	C110623	C_5_H_10_O	86.1	692.2	231.425	1.42491	Fermented bread, fruity, nuts
66	2-Methylbutanal	C96173	C_5_H_10_O	86.1	687.5	228.314	1.39665	Cocoa
67	1,2-Dimethoxyethane	C110714	C_4_H_10_O_2_	90.1	655.8	213.273	1.29706	-
68	1-Propanethiol	C107039	C_3_H_8_S	76.2	613.7	194.821	1.17255	Cabbage, sweet onion
69	Ethyl acetate	C141786	C_4_H_8_O_2_	88.1	601.2	189.644	1.10211	Fruity
70	2-Butanone	C78933	C_4_H_8_O	72.1	583.3	182.487	1.24177	Etheric, diffusive, slightly fruity
71	Butanal	C123728	C_4_H_8_O	72.1	574.3	178.999	1.28661	Chocolate
72	2-Butanol	C78922	C_4_H_10_O	74.1	614.6	195.193	1.32263	Sweet apricot
73	2-Propanone	C67641	C_3_H_6_O	58.1	487.9	148.638	1.11428	Apple pear
74	Propanal	C123386	C_3_H_6_O	58.1	488.3	148.782	1.15269	Whiskey, cocoa nuts
75	Methyl acetate	C79209	C_3_H_6_O_2_	74.1	483.7	147.297	1.19243	Fruity
76	Ethyl formate	C109944	C_3_H_6_O_2_	74.1	535.7	164.731	1.22221	Fermentation, wine
77	3-Methylpentanoic acid	C105431	C_6_H_12_O_2_	116.2	956.6	540.777	1.60365	Acidic cheesy flavor
78	γ-Valerolactone	C108292	C_5_H_8_O_2_	100.1	933.4	500.594	1.13124	Herbaceous
79	Ethyl 3-oxobutanoate	C141979	C_6_H_10_O_3_	130.1	922.2	482.244	1.60577	-
80	1-Propanol	C71238	C_3_H_8_O	60.1	555.2	171.805	1.11225	Alcoholic, fermented flavor
81	3-Ethylpyridine	C536787	C_7_H_9_N	107.2	962.1	550.747	1.52004	Tobacco
82	Cyclohexanone	C108941	C_6_H_10_O	98.1	897.7	444.525	1.16542	Minty flavor

Note: CAS #—CAS Registry Number; MW—Molecular weight; RI—Retention index; Rt—Retention time; Dt—Drift time; M and D—Monomer and dimer; “-”—Aroma characteristics not found on https://thegoodscentscompany.com/ (accessed on 11 May 2025).

## Data Availability

The original contributions presented in this study are included in the article. Further inquiries can be directed to the corresponding authors.

## References

[1] Xu C., Xie Q., Kuo C.-L., Yang X., Huang D. (2025). Evidence-Based Nutraceuticals Derived from *Antrodia cinnamomea*. Foods.

[2] Lin Z.-H., Phan S.-N.-C., Tran D.-N.-H., Lu M.-K., Lin T.-Y. (2025). Anti-Inflammatory and Anticancer Effects of Polysaccharides from *Antrodia cinnamomea*: A Review. J. Chin. Med. Assoc..

[3] Ming Y., Li Y., Chu J., Zhou X., Huang Y., Yang S., Mu Y., Wang L., Zhang R., Cheng X. (2024). Comparative Analysis of Metabolites and In Vitro Hypoglycemic Activity of *Taiwanofungus camphoratus* Cultured Using Various Methods. Appl. Biol. Chem..

[4] Liu Q., Qiang S., Tang J., Dai J., Liu B., Ye Q., Li H. (2025). Research Progress on the Antibacterial Properties of *Antrodia cinnamomea* and Its Host Against Foodborne Pathogens. Food Ferment. Ind..

[5] Chu J., Ming Y., Cui Q., Zheng N., Yang S., Li W., Gao H., Zhang R., Cheng X. (2022). Efficient Extraction and Antioxidant Activity of Polyphenols from *Antrodia cinnamomea*. BMC Biotechnol..

[6] Xia Y., Li W., Xu G. (2011). Analysis of Active Metabolites in Solid-State Fermentation Products of *Antrodia cinnamomea*. Food Ferment. Ind..

[7] Dudekula U.T., Doriya K., Devarai S.K. (2020). A Critical Review on Submerged Production of Mushroom and Their Bioactive Metabolites. Biotech.

[8] Dai J., Liu B., Ji D., Yuan L., Zhou W., Li H. (2023). Extraction, Isolation, Identification, and Bioactivity of Polysaccharides from *Antrodia cinnamomea*. Qual. Assur. Saf. Crops Foods.

[9] Jiang H., Duan W., Zhao Y., Liu X., Wen G., Zeng F., Liu G. (2023). Development of a Flavor Fingerprint Using HS-GC-IMS for Volatile Compounds from Steamed Potatoes of Different Varieties. Foods.

[10] Tatli S., Mirzaee-Ghaleh E., Rabbani H., Karami H., Wilson A.D. (2022). Prediction of Residual NPK Levels in Crop Fruits by Electronic-Nose VOC Analysis Following Application of Multiple Fertilizer Rates. Appl. Sci..

[11] Hernández-Mesa M., Escourrou A., Monteau F., Le Bizec B., Dervilly-Pinel G. (2017). Current Applications and Perspectives of Ion Mobility Spectrometry to Answer Chemical Food Safety Issues. Trends Anal. Chem..

[12] Perl T., Jünger M., Vautz W., Nolte J., Kuhns M., Borg-von Zepelin M., Quintel M. (2011). Detection of Characteristic Metabolites of Aspergillus Fumigatus and Candida Species Using Ion Mobility Spectrometry—Metabolic Profiling by Volatile Organic Compounds: Fungal Volatile Metabolites. Mycoses.

[13] Chang M., Liu Y., Li Z., Feng X., Xiao Y., Huang W., Liu Y. (2024). Fingerprint Analysis of Volatile Flavor Compounds in Twenty Varieties of Lentinula Edodes Based on GC-IMS. Sci. Hortic..

[14] Ge Y., Wang L., Huang Y., Jia L., Wang J. (2024). Characteristic Flavor Compounds in Guizhou Green Tea and the Environmental Factors Influencing Their Formation: Investigation Using Stable Isotopes, Electronic Nose, and Headspace-Gas Chromatography Ion Migration Spectrometry. LWT.

[15] Duan Z., Dong S., Dong Y., Gao Q. (2021). Geographical Origin Identification of Two Salmonid Species via Flavor Compound Analysis Using Headspace-Gas Chromatography-Ion Mobility Spectrometry Combined with Electronic Nose and Tongue. Food Res. Int..

[16] Gerhardt N., Birkenmeier M., Sanders D., Rohn S., Weller P. (2017). Resolution-Optimized Headspace Gas Chromatography-Ion Mobility Spectrometry (HS-GC-IMS) for Non-Targeted Olive Oil Profiling. Anal. Bioanal. Chem..

[17] Cavanna D., Zanardi S., Dall’Asta C., Suman M. (2019). Ion Mobility Spectrometry Coupled to Gas Chromatography: A Rapid Tool to Assess Eggs Freshness. Food Chem..

[18] Xia Y., Zhang B., Li W., Xu G. (2011). Changes in Volatile Compound Composition of *Antrodia camphorata* during Solid State Fermentation: Volatile Compound Composition of *Antrodia camphorata* during Fermentation. J. Sci. Food Agric..

[19] Liu H., Jia W., Zhang J., Pan Y. (2008). GC-MS and GC-Olfactometry Analysis of Aroma Compounds Extracted from Culture Fluids of *Antrodia camphorata*. World J. Microbiol. Biotechnol..

[20] He Z., Lu Z., Xu H., Shi J., Xu Z. (2011). Determination of Volatile Compounds in *Antrodia cinnamomea* Mycelia by Headspace Solid-Phase Microextraction-Gas Chromatography-Mass Spectrometry. Chin. J. Med. Mater..

[21] Sebzalli Y.M., Wang X.Z. (2001). Knowledge Discovery from Process Operational Data Using PCA and Fuzzy Clustering. Eng. Appl. Artif. Intell..

[22] Buratti S., Malegori C., Benedetti S., Oliveri P., Giovanelli G. (2018). E-Nose, e-Tongue and e-Eye for Edible Olive Oil Characterization and Shelf Life Assessment: A Powerful Data Fusion Approach. Talanta.

[23] Jin W., Zhao S., Sun H., Pei J., Gao R., Jiang P. (2023). Characterization and Discrimination of Flavor Volatiles of Different Colored Wheat Grains after Cooking Based on GC-IMS and Chemometrics. Curr. Res. Food Sci..

[24] Li Y., Yuan L., Liu H., Liu H., Zhou Y., Li M., Gao R. (2023). Analysis of the Changes of Volatile Flavor Compounds in a Traditional Chinese Shrimp Paste during Fermentation Based on Electronic Nose, SPME-GC-MS and HS-GC-IMS. Food Sci. Hum. Wellness.

[25] Moses T., Pollier J., Thevelein J.M., Goossens A. (2013). Bioengineering of Plant (Tri)Terpenoids: From Metabolic Engineering of Plants to Synthetic Biology In Vivo and In Vitro. New Phytol..

[26] Murakami Y., Kawata A., Suzuki S., Fujisawa S. (2018). Cytotoxicity and Pro-/Anti-Inflammatory Properties of Cinnamates, Acrylates and Methacrylates against RAW264.7 Cells. Vivo.

[27] Wang H., Li X., Wang J., Vidyarthi S.K., Wang H., Zhang X.-G., Gao L., Yang K.-W., Zhang J.-S., Xiao H.-W. (2022). Effects of Postharvest Ripening on Water Status and Distribution, Drying Characteristics, Volatile Profiles, Phytochemical Contents, Antioxidant Capacity and Microstructure of Kiwifruit (Actinidia Deliciosa). Food Control.

[28] Deveci E., Tel-Cayan G., Emin Duru M., Turkoglu A. (2017). Characterization of Aromatic Volatile Compounds of Eight Wild Mushrooms by Headspace GC-MSD. Chem. Nat. Compd..

[29] Lu J., Lin X., Zhang R., Wu J., Dai Q., Luo D., Li L., Chen X., Huang G. (2015). Roger RUAN Analysis of Aroma Components in American Almonds by HS-SPME-GC-MS. Food Sci..

[30] Han X., Guan Q., Liu X. (2024). Analysis of Volatile Flavors and Non-Volatile Taste Compounds in Seven Common Edible Fungi. Food Technol..

[31] Li H., Xi B., Lin S., Tang D., Gao Y., Zhao X., Liang J., Yang W., Li J. (2024). Volatile Flavor Analysis in Yak Meat: Effects of Different Breeds, Feeding Methods, and Parts Using GC-IMS and Multivariate Analyses. Foods.

[32] Zhang P.P., Gui X.J., Fan X.H., Li H.Y., Li X.P., Dong F.Y., Wang Y.L., Shi J.H., Liu R.X. (2025). Quality Identification of Amomi Fructus Using E-Nose, HS-GC-IMS, and Intelligent Data Fusion Methods. Front. Chem..

[33] Liu L., Zuo Z.T., Wang Y.Z., Xu F.R. (2020). A Fast Multi-Source Information Fusion Strategy Based on FTIR Spectroscopy for Geographical Authentication of Wild Gentiana Rigescens. Microchem. J..

